# Perturbation of water‐equivalent thickness as a surrogate for respiratory motion in proton therapy

**DOI:** 10.1120/jacmp.v17i2.5795

**Published:** 2016-03-08

**Authors:** Jason E. Matney, Peter C. Park, Heng Li, Laurence E. Court, X. Ron Zhu, Lei Dong, Wei Liu, Radhe Mohan

**Affiliations:** ^1^ Department of Radiation Oncology University of North Carolina Cancer Hospital Chapel Hill NC; ^2^ Department of Radiation Physics University of Texas MD Anderson Cancer Center Houston TX; ^3^ Department of Radiation Oncology Mayo Clinic Arizona Scottsdale/Phoenix AZ; ^4^ Department of Medical Physics Scripps Proton Therapy Center San Diego CA USA

**Keywords:** proton therapy, respiratory motion, water‐equivalent thickness, water‐equivalent path length

## Abstract

Respiratory motion is traditionally assessed using tumor motion magnitude. In proton therapy, respiratory motion causes density variations along the beam path that result in uncertainties of proton range. This work has investigated the use of water‐equivalent thickness (WET) to quantitatively assess the effects of respiratory motion on calculated dose in passively scattered proton therapy (PSPT). A cohort of 29 locally advanced non‐small cell lung cancer patients treated with 87 PSPT treatment fields were selected for analysis. The variation in WET (ΔWET) along each field was calculated between exhale and inhale phases of the simulation four‐dimensional computed tomography. The change in calculated dose (ΔDose) between full‐inhale and full‐exhale phase was quantified for each field using dose differences, 3D gamma analysis, and differential area under the curve (ΔAUC) analysis. Pearson correlation coefficients were calculated between ΔDose and ΔWET. Three PSPT plans were redesigned using field angles to minimize variations in ΔWET and compared to the original plans. The median ΔWET over 87 treatment fields ranged from 1‐9 mm, while the ΔWET 95th percentile value ranged up to 42 mm. The ΔWET was significantly correlated (p<0.001) to the ΔDose for all metrics analyzed. The patient plans that were redesigned using ΔWET analysis to select field angles were more robust to the effects of respiratory motion, as ΔAUC values were reduced by more than 60% in all three cases. The tumor motion magnitude alone does not capture the potential dosimetric error due to respiratory motion because the proton range is sensitive to the motion of all patient anatomy. The use of ΔWET has been demonstrated to identify situations where respiratory motion can impact the calculated dose. Angular analysis of ΔWET may be capable of designing radiotherapy plans that are more robust to the effects of respiratory motion.

PACS number(s): 87.55.‐x

## I. INTRODUCTION

A potential benefit of proton radiotherapy is that protons exhibit a dose distribution with a finite range in a medium, unlike that of photons. Protons exhibit their highest linear energy transfer near the end of range in a medium, which is known as the Bragg peak. Beyond the Bragg peak, virtually no dose is deposited. Therefore, the finite range of protons in tissue can be used to spare dose to normal tissues distal to the tumor. However, the depth of penetration is dependent on the accurate knowledge of the tissue densities along the beam path. One major source of intrafractional tissue density variations along the beam path is respiratory motion.^(1)^ Respiratory motion is patient‐specific^(2)^ and individual respiratory characteristics can vary in period, amplitude, and regularity during observation.^(3)^ Furthermore, respiratory motion patterns can vary between fractions^4^, ^5^ and over the course of radiotherapy the tumor may change in mobility as well as size and shape.^(6)^ With the wide use of four‐dimensional computed tomography (4D CT) in radiotherapy clinics, direct measurement of tumor motion magnitude is possible. The tumor motion magnitude estimated from 4D CT can be used as the first estimate of the dosimetric influence of breathing motion. For example, the report of American Association of Medical Physicists Task Group 76 recommends that the motion magnitude greater than 5 mm as the threshold recommended for motion management protocols such as gating or breath‐hold techniques.^(7)^ It is widely held that respiratory‐induced dose perturbation will be greater for proton therapy than for conventional photon therapy.^(8)^ Previous studies^9^, ^10^ have demonstrated that variation in dose due to respiratory motion was not predicted by the extent of tumor motion.

Quality assurance and beam commissioning data for proton therapy are commonly measured in water‐equivalent material.^(11)^ For convenience of relating proton ranges in different materials, the proton beam energy can be described in terms of water‐equivalent thickness (WET). Therefore, we quantify the range of the proton beam not only with a physical depth of penetration in a given medium, but with WET that the proton beam would penetrate. Accurate knowledge of the patient anatomy is required to calculate the physical proton range.

Mori et al.^(12)^ reported that the WET fluctuations during cardiac motion could be used to assess heavy charged particle range fluctuations. In a follow‐up study, Mori et al.^(13)^ suggested the use of WET analysis of 4D CT data to quantify changes in lung tissue to optimize gated planning and delivery of lung tumors. Our study expands upon this idea to demonstrate the efficacy of WET analysis as a surrogate of tumor motion. In this work, we directly compare the respiratory motion‐induced change in WET due to the variation of the TPS planned dose at inhale and exhale phases of respiration. The purpose of this study was to propose the use of ΔWET as a metric for quantifying respiratory motion of patient anatomy during proton therapy.

## II. MATERIALS AND METHODS

### A. Patient selection

For this work, we analyzed patient data from 29 PSPT plans from patients enrolled in an institutional review board approved trial for locally advanced stage II ‐ IIIB non‐small cell lung cancer. Patient information is given in Table 1. All patients on the trial had PSPT plans approved for treatment by board‐certified physicians. Plans were designed following the methodology of Moyers et al.^(14)^ and Kang et al.^(15)^ Field apertures were designed to project outside of the ITV by a distance that accounts for setup uncertainty and the 90%‐50% penumbra width. To select spread‐out Bragg peak width for each field, proximal and distal margins were added to the ITV. Field compensators were designed following the methodology of Engelsman et al.^(16)^ to ensure target coverage during respiration. Target and normal tissue contours were delineated on the maximum‐intensity‐projection and average of the 4D CT phases, respectively. Plan dose was calculated on the average 4D CT dataset using the Eclipse version 8.1 treatment planning system (Varian Medical Systems, Palo Alto, CA). A total number of 87 fields were used for the 29 PSPT plans, averaging three fields per patient.

**Table 1:** Treatment plan overview. Treatment field data from 29 patients were collected to correlate WET to the dosimetric effects of respiratory motion. The absolute displacement of the tumor centroid during respiration between inhale and exhale phase of the 4D CT is labeled as “motion.”

*Patient #*	*Motion (mm)*	*Rx (Gy)*	*Location*	*ITV(cc)*	*Fields/Plan*
1	3	74	RUL	786	5
2	4	74	RUL	510	3
3	5	74	RUL	574	4
4	6	74	LUL	308	3
5	6	74	RUL	1205	2
6	6	74	RUL	162	3
7	7	66	RUL	625	2
8	7	66	LLL	343	5
9	7	74	LLL	314	3
10	8	66	RUL	630	3
11	8	74	LLL	247	3
12	8	74	RUL	249	3
13	9	74	RLL	311	2
14	9	66	RLL	239	3
15	9	74	RLL	168	3
16	10	66	RLL	493	3
17	12	74	RUL	656	3
18	12	60	LUL	264	3
19	13	74	RLL	213	3
20	17	74	RLL	212	2
21	9	74	LUL	79	2
22	9	66	RUL	132	3
23	10	74	LLL	103	3
24	9	60	LUL	146	3
25	11	74	RUL	293	2
26	6	74	LLL	41	3
27	4	66	RLL	239	3
28	5	74	RUL	196	4
29	8	74	LLL	225	3

Rx=prescription dose; ITV=internal target volume; RLL=right lower lobe; RUL=right upper lobe; LLL=left lower lobe; LUL=left upper lobe.

### B. WET analysis

Software was designed using MATLAB (MathWorks, Natick, MA) to analyze the WET variance between the inhale and exhale anatomical state of the patient. First, the software imports the exhale (T50) and inhale (T0) 4D CT images acquired at initial simulation. The user is prompted to select the tumor target from the contoured structures in the treatment plan. For this study, the internal target volume (ITV) that encompasses the position of clinical target volume (CTV) at all 10 phases of 4D CT dataset was used. To perform WET analysis as a function of field angle, the software prompts the user to input the desired field angle spacing. For a given beam angle, the program created a series of rays along the beam's eye view (BEV), ranging to the distal end of the ITV. In this study, because we are calculating the range to reach a geometric point in the patient (e.g., distal surface of the target), the WET calculations ignore any effects of multiple Coulomb scattering.

The program then calculated a matrix of the WET values corresponding to the projected CT voxel size (1×1×2.5 mm) perpendicular to the field direction. Each element (or pixel) of the WET matrix represents the required proton WET to sufficiently cover the ITV. The change in water‐equivalent thickness during respiration (ΔWET) along each ray to the distal side of the target was calculated by taking the difference between the WET matrices generated from the T50 and T0 phases of 4D CT. The T50 and T0 phases were chosen to analyze in order to find the largest expected dosimetric effects caused by respiratory motion. This process was repeated for a series of coplanar fields specified by the user‐defined angular spacing over a 360° arc around the patient. No noncoplanar field angles were used in the patient cohort, thus were not analyzed.

**Figure 1:**
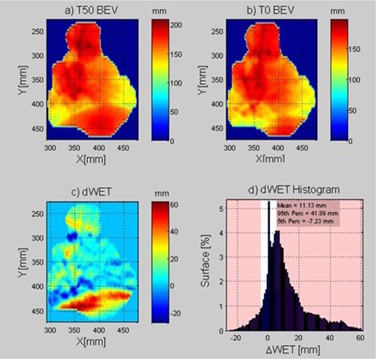
Example output from the ΔWET analysis software for an example treatment field at 250°. On the top row is the beam's‐eye view WET depth matrix to the distal edge of the target for T50 (a) and T0 (b). The ΔWET matrix (c) is calculated from the difference of the first two images. To visually assess the ΔWET per field, a histogram (d) is shown of ΔWET.

**Figure 2:**
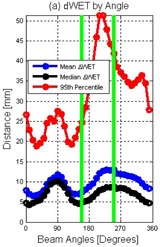
An example of the ΔWET software output of the mean, median, and 95th percentile of the absolute value of every 10° in a coplanar arrangement around a patient. Green lines are added to illustrate the two clinical field angles selected for PSPT treatment prior to ΔWET analysis.

Statistics (e.g., mean, median, 5^th^, and 95th percentiles) of the ΔWET matrix were calculated for each field angle. An example of the ΔWET analysis program output for a single field angle is shown in Fig. 1. In Fig. 2, an example of the calculated ΔWET for each field was plotted as a function of angle around the patient. For all patients listed in Table 1, each treatment field was analyzed using the ΔWET program and two metrics were reported for each of the 87 planned treatment fields: the median and 95th percentile of ΔWET.

### C. Dose analysis

To determine whether ΔWET can predict dosimetric changes due to respiration, this work correlated the ΔWET to a respiratory‐induced deviation in calculated dose. This required calculation of dose on a field‐by‐field basis. The dose from each field for each treatment plan was recalculated on the T50 and T0 phases of simulation dataset using the Eclipse treatment planning software. The resulting T50 and T0 dose matrices for each individual field were exported for analysis in the Computational Environment for Radiotherapy Research (CERR) software platform.^(17)^

For plans with multiple fields, the field weighting factors were not necessarily equal. At the discretion of the treatment planner, some fields were weighted more or less to meet target and normal tissue goals. To compare the changes in dose between fields with different weighting, the dose was normalized to remove the weighting such that the dose delivered by each field achieved the full prescription dose. The normalized dose differences between the T50 and T0 phases were calculated using the Eclipse TPS for all planned treatment fields. Four metrics were identified to compare the change in dose during respiration, or ΔDose, values: 1) root mean square deviation (RMSD), 2) histogram ±3% dose passing rate, 3) 3D gamma analysis, and 4) differential area under the DVH curve.

The first method was to calculate the root mean square deviation of the dose difference. For all voxels within the external body contour of the patient, a MATLAB routine was written to read the normalized T50 and T0 field doses, ignoring any voxels outside the patient body (e.g., dose calculated in the treatment couch), and calculate the RMSD between the two dose clouds.

The second method was a histogram analysis of the dose difference between the T50 and T0 dose cloud. A dose difference tolerance of 3% of prescription was selected arbitrarily as the passing criteria. To compare the changes in only the irradiated volumes of the patient, we excluded all voxels that received less than 3% of the prescription dose. After removing the uninvolved voxels, if the change in voxel dose between the T50 and T0 dose was within ±3% of the prescription dose, the voxel was considered “passing.” The next method used gamma analysis^(18)^ in three dimensions to quantify the differences in the T50 and T0 dose cloud.^(19)^ We chose 3% dose difference and 3 mm distance to agreement criteria with a 3% low dose threshold following the original 3D gamma analysis report by Wendling et al.^(19)^

The last method used to evaluate ΔDose was the differential area under the cumulative T0 and T50 DVH curve (ΔAUC).^(20)^ For a particular structure, the ΔAUC can be expressed by the equation:

(1) $$\Delta AUC=\sum_{D}(\left|DVH_{T50}(D)-DVH_{T0}(D)\right|*d)$$

where *DVHT_50_(D)* and *DVH_T0_(D)* represent the value of the cumulative DVH for T50 and T0, respectively, for a given dose bin D with width *d*. A larger ΔAUC value for a structure indicates a greater change in dose received during respiration for that particular structure. For each field, the PTV, total lung, esophagus, heart, and spinal cord ΔAUC values were calculated between the normalized T50 and T0 field doses. For the purpose of simplifying the analysis of a given plan, the ΔAUC of the PTV, total lung, esophagus, heart, and spinal cord were combined to give single metric called “total ΔAUC”.

### D. Correlation of ΔWET to ΔDose

Linear regression fits to the data were calculated between the ΔWET and ΔDose metrics analyzed. To reduce type 1 error rate (false‐positive) inflation due to multiple hypothesis testing, we conservatively adjusted our significant p‐value with a Bonferroni correction^(21)^ by the number of hypotheses (12) to p=0.05/12 approximately equal to 0.004.

### E. ΔWET reduction treatment planning

In Fig. 2, it can be observed that the angles selected for treatment (green lines) could be altered to reduce the values of ΔWET. Analysis of ΔWET could be a clinically useful tool to select field angles that would reduce the impact of respiratory motion on the PSPT plan. The current method to select field angles is currently through a process of trial and error. The assessment of the effect of respiratory motion on a proton plan is only conducted after the treatment plan has been designed.

As a demonstration of the potential use of ΔWET analysis in proton therapy planning, this work has identified three PSPT plans from our cohort with the largest variations between T0 and T50 calculated dose. This work sought to minimize the effect of respiratory motion on the planned dose by selecting new beam angles using the ΔWET analysis program.

Figure 2 shows an example where the clinically chosen fields (green lines) were not necessarily the lowest values of ΔWET. We attempted to redesign this plan using the ΔWET analysis results as guidance. The fields that were selected to optimize the ΔWET values for this particular patient were 155° and 350°. To redesign the plan with the new angles, we followed the same design methodology as in the original plan; the same methodologies were used to calculate new proximal and distal margins, apertures, compensators, and energy selections. Once the new “ΔWET reduced plan” was completed, the plan dose was recalculated on the T0 and T50 datasets using Eclipse TPS. RMSD and ΔAUC analysis were used to compare the changes in T50 and T0 calculate dose between the original plan and the ΔWET analysis replan.

## III. RESULTS

**Figure 3:**
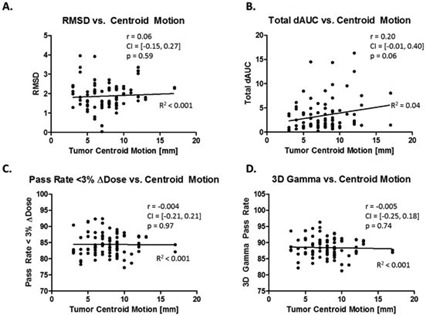
Plots of ΔDose for the 87 normalized field doses were plotted against the tumor centroid motion observed for each patient. Pearson correlation coefficients (*r*), 5%‐95% confidence intervals, and p‐values of the correlation coefficient were calculated between ΔDose metrics and the tumor motion.

The ΔWET and ΔDose metrics have been calculated for 87 treatment fields over 29 PSPT plans. In Fig. 3, the ΔDose metrics for 87 fields over 29 patients were plotted against the current clinical metric for assessing respiratory motion; tumor centroid motion measured between the T50 and T0 images.^(22)^ It was observed that the ΔDose metrics demonstrated no significant correlation to the tumor centroid motion; R^2^ was near zero in all cases with the largest R^2^ value being 0.04.

**Figure 4:**
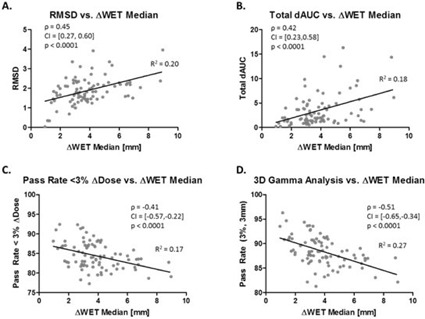
Relationship between ΔWET median and ΔDose displayed in the same fashion as Fig. 3 for 87 treatment fields.

Unlike the tumor centroid motion metric, a significant correlation was found between median ΔWET values and all four ΔDose metrics, as shown in Fig. 4. The RMSD (Fig. 4(a)) and the ΔAUC (Fig. 4(b)) were positively correlated to the median ΔWET, while the percentage of irradiated volumes within ±3% dose agreement (Fig. 4(c)) and 3D gamma pass rate (Fig. 4(d)) between the T0 and T50 respiratory states were negatively correlated to the median ΔWET. The $R^{2}$ values of these metrics with ΔWET were between 0.17 and 0.27. This was an improvement compared to tumor centroid motion (Fig. 3).

**Figure 5:**
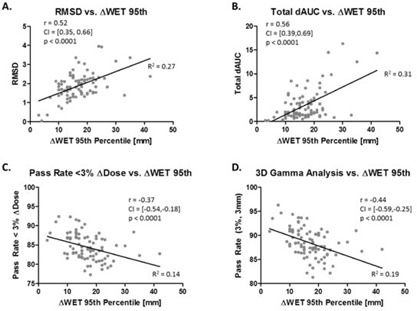
Relationship between ΔWET 95th percentile and ΔDose displayed in the same fashion as Fig. 3 for 87 treatment fields.

In Fig. 5, the ΔWET 95th percentile values were compared against the four ΔDose metrics for each treatment field in the cohort. As in the previous figure, ΔWET was significantly (p<0.0001) correlated to each ΔDose metric analyzed.

The investigated ΔWET metrics were found to have moderate (0.37<|r|<0.56), but significant (p≥0.0001) correlation to the four ΔDose metrics. This suggests that ΔWET provides a metric that is correlated to the effects of respiratory motion on the planned dose, while the tumor centroid motion alone is not correlated.

**Figure 6:**
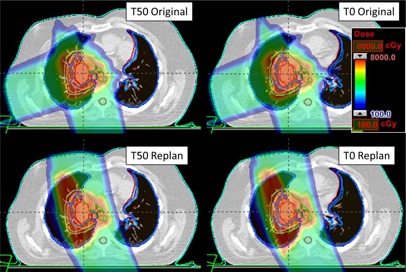
An example of the ΔWET guided plan (bottom) compared to the original, clinical plan (top). An axial slice plan dose is shown on the top for T50 (left) and T0 (right) image set. The ΔWET guided plan has new field angles of 155° and 350° which were determined using the ΔWET analysis program.

Three patients were identified for replanning efforts using the ΔWET analysis to select new field angles. In Fig. 6, the calculated dose delivered during exhale and inhale for the plans containing two new field angles (bottom, 155° and 350°) compared to the original angles (top, 165° and 250°).

**Figure 7:**
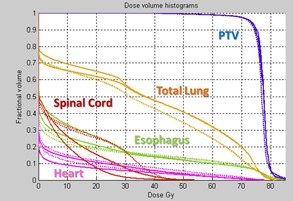
For the example patient in Fig. 6, the original plan T50 and T0 DVH curves (solid lines) are shown in comparison to the ΔWET reduced plan T50 and T0 (dotted lines).

In Fig. 7, the DVH from the two plans demonstrated in Fig. 6 is shown. The solid lines show the original plan's DVH curves for T50 and T0, while the dotted lines shown the ΔWET reduction plan T50 and T0 DVH curves. The goal of creating the ΔWET reduction plans was not necessarily to reduce the normal tissue dose, but to reduce the variation in dose delivered between exhale and inhale respiratory phases. While the cord dose was increased by the new plan, it still respected all clinical OAR constraints. In the ΔWET‐guided replan, the variation of dose between the T50 and T0 phase of respiration was reduced for all OAR volumes analyzed.

For the three sets of original and ΔWET reduction plans, the variation between T50 and T0 plan dose was compared using ΔAUC and RMSD metrics. Table 2 lists the results for the comparison between the original plan and the ΔWET reduction plan. For these three plans that were redesigned using ΔWET analysis, the RMSD between the T50 and T0 dose cloud was reduced by ∼15%−35%. The ΔAUC value was reduced by more than 60% in the ΔWET reduction plan compared to the original plan. By demonstrating the calculated dose changes less between inhale and exhale, these results demonstrate that the ΔWET reduction plans are more robust to the effects of respiratory motion compared to the original treatment plan.

**Table 2:** ΔWET guided plan design. Three PSPT plans (A‐C) were redesigned using field angles that improved upon the ΔWET metrics are compared to the original plan (Original). Between the T0 and T50 dose clouds for both plans, the root mean square deviation (RMSD) was calculated. For both the original and ΔWET plan, the T50 and T0 DVH curves were compared for each plan using ΔAUC analysis

*Patient*	*Plan*	*RMSD*	*PTV ΔAUC*	*Lung ΔAUC*	*Eso ΔAUC*	*Heart ΔAUC*	*Spine ΔAUC*	*Total ΔAUC*
A	Original	2.24	0.34	0.47	3.11	3.59	0.00	7.50
A	ΔWET	1.46	0.18	1.06	0.13	0.02	0.04	1.43
B	Original	2.03	0.25	1.62	1.93	1.55	2.22	7.57
B	ΔWET	1.45	0.06	1.49	0.61	0.15	0.59	2.90
C	Original	2.23	0.17	1.91	1.17	1.61	2.25	7.11
C	ΔWET	1.91	0.08	0.70	0.10	1.51	0.26	2.65

## IV. DISCUSSION

We have introduced the use of a new metric to identify the impact of respiratory motion in proton therapy: the change in water‐equivalent thickness during respiration, or ΔWET. The traditionally used metric of tumor motion describes only a small subregion of the overall patient anatomy. The lack of correlation between ΔDose and tumor centroid motion reaffirms studies such as the one by Dowdell et al.^(10))^ that state that the displacement of the tumor does not predict the effects of respiratory motion on the calculated dose in proton therapy. A topical review by Bert et al.^(1)^ outlined that for scanned beam proton therapy, the effects of respiratory motion would be even greater. It should be noted that the current study was limited to only passively scattered delivery. While the authors believe that the magnitude of tumor motion is still an important metric to be evaluated, respiratory management decisions based on the tumor motion alone should be avoided in proton therapy.

The results demonstrate that there is no threshold of single field ΔWET that strongly correlates to an acceptable or unacceptable level of respiratory‐induced dosimetric change in a multifield plan. Respiration is a complex process, typically quantified only on the tumor motion between inhale and exhale. Published results have pointed out the shortcomings in such methods, and especially in proton therapy. We have shown the tumor motion was not significantly correlated to dosimetric change due to respiration, so we think it is of interest that the metric of ΔWET shows moderate correlation.

It is known that the respiratory tumor motion can vary between fractions of treatment, and this is a limitation of the current study. This study did not consider 4D dose calculation as this would require the use of deformable image registration, which would add additional uncertainty. We plan follow‐up studies to identify the variation in ΔWET during the course of therapy using weekly 4D CT scans. Another important topic for follow‐up studies is to correlate ΔWET to ΔDose during scanned beam proton therapy. Such work will require 4D dose calculation for scanned beam therapy.^23^, ^24^

The calculation of WET thickness as outlined above is a relatively fast process, taking less than a second per field angle on a 3.3 GHz Intel Xeon processor. An entire arc can be analyzed with a fine field interval (10°) in roughly a minute. We mention the speed of calculation to show that this method does not require long calculation times, as Monte Carlo methods may, and would not delay the start of treatment planning if used to suggest initial beam angles to treatment planners. This work only considered a coplanar beam arrangement; however, the ΔWET analysis method is extendable to search for any allowed beam geometry. We anticipate that future additions to the field angle selection program will avoid field angles in which the beam would pass through critical OARs.

It should be noted that the dose‐volume indices in the ΔWET‐reduced plan were not always improved compared to the original plan. However, the variation in planned dose between the T50 and T0 respiratory phase was reduced in all three cases. Our intention was not necessarily to create a superior plan compared to the original, but to create a plan that was more robust to the effects of respiratory motion. It is important to note that there are many other factors that are considered when selecting field angles for PSPT. Respiratory motion is only one factor among many to consider; however, ΔWET analysis provides guidance in field angle selection. For example, in Fig. 2, large ΔWET variations were observed for field angles around 270°. The WET variations proximal to the target were caused by diaphragm motion affecting fields near a 270° angle. In this example, the use of ΔWET analysis would have quickly alerted the planning team that respiratory motion would greatly impact the calculated dose distribution of the initially chosen field angles. These results show that selecting field angles that minimize ΔWET variations can help design plans that achieve plans that are more robust to the effects of respiratory motion

It was our intent to create a useful clinical tool that could be applied before any fields were chosen for a plan. The ΔWET analysis suggested in this paper only requires 4D CT and structural information on the treatment target. Delineation of target volumes by the physician is one of the first steps in creating the treatment plan. Therefore, ΔWET analysis can be completed early in the treatment planning process to guide beam angle selection or suggest motion mitigated techniques such as breath‐hold or gating, if necessary. It should be noted that selected angles may help minimize variation in the proximal and distal margins; the lateral margins would be unaffected.

The results suggest that the effects of respiratory motion on the planned dose should be considered on a field‐specific basis. In the current methodology of PSPT planning, treatment fields are designed by assigning margins on a field‐by‐field basis. If we account for uncertainties on a field‐specific basis, it would follow that we should assess uncertainties such as dose variation due to respiration motion on a field‐specific basis. In general, the largest contribution to WET variation was observed to be the diaphragm motion for lower lobe tumors.

If a particular field is the cause of large dose variations during respiration, it could be advantageous to select a different angle, or reduce the offending field's weight. However, we are not able to make recommendations for patient‐specific implementations of this work at this time. From this work, it was observed that ΔWET analysis could suggest field angles that minimized ΔWET to produce PSPT plans that were more robust to the effects of respiratory motion.

## V. CONCLUSIONS

In this work, we have investigated a new metric to quantify the impact of respiratory motion that incorporates global tissue variation during respiration: ΔWET. This metric is of particular interest in proton therapy, where the range of the proton can be stated in terms of water‐equivalent thickness. This work has led to the development of a process that can analyze the WET between the inhale and exhale phase of respiration. This work has demonstrated that the ΔWET metric was significantly correlated to various metrics that quantified that change in planned dose between exhale and inhale phases of respiration. Incorporating ΔWET analysis to guide field angle selection produced plans that were more robust to the effects of respiratory motion.

## COPYRIGHT

This work is licensed under a Creative Commons Attribution 4.0 International License.
